# Insights into binding of S100 proteins to scavenger receptors: class B scavenger receptor CD36 binds S100A12 with high affinity

**DOI:** 10.1007/s00726-016-2349-2

**Published:** 2016-10-12

**Authors:** Christoph Tondera, Markus Laube, Jens Pietzsch

**Affiliations:** 1 Department of Radiopharmaceutical and Chemical Biology, Institute of Radiopharmaceutical Cancer Research, Helmholtz-Zentrum Dresden-Rossendorf, Dresden, Germany; 2 Department of Chemistry and Food Chemistry, Technische Universität Dresden, Dresden, Germany

**Keywords:** EF-hand calcium-binding proteins, Surface plasmon resonance, Pattern recognition receptors, Damage-associated molecular patterns, Receptor for advanced glycation endproducts (RAGE)

## Abstract

The EF-hand type calcium-binding protein S100A12 exerts numerous intra- and extracellular functions of (patho)physiological relevance. Therefore, receptors of S100A12 are of high interest for research and clinical applications. Beside the extensively studied receptor for advanced glycation endproducts (RAGE), G-protein coupled receptors and more recently, scavenger receptors are suggested to be putative S100A12 receptors. Own findings and further information from the literature predestined CD36, a class B scavenger receptor, as promising candidate. To substantiate or prove against this hypothesis, this study aimed at investigation of interaction of S100A12 and CD36 on molecular and cellular level by the use of surface plasmon resonance (SPR), radio- and fluorescence-tracer-based cell binding, and cell activation experiments. S100A12 revealed binding affinity to CD36 in the low nanomolar range, essentially, at the CD36 thrombospondin-1 binding site. Additionally, S100A12-mediated translocation of CD36 to the membrane and elevation of both CD36 and peroxisome proliferator-activated receptor γ (PPARγ) expression was observed, which suggest a potential regulatory function of S100A12–CD36 interaction.

**Electronic supplementary material:**

The online version of this article (doi:10.1007/s00726-016-2349-2) contains supplementary material, which is available to authorized users.

## Introduction

S100A12 is a member of the S100 protein family comprising more than 20 calcium-binding proteins of the EF-hand type. Most of them are small (molecular weight <13 kDa) distinctly homoacidic proteins (Donato et al. 2013), which regulate numerous intracellular and/or extracellular functions, including activation of enzymes, maintenance of calcium homeostasis, interaction with cytoskeletal components, and interaction with receptors (Vogl et al. 1999; Hatakeyama et al. 2004; Donato et al. 2013). Once released into the extracellular space or the circulating blood, mostly by granulocytes, S100A12 in a cytokine-like manner exerts proinflammatory effects, e.g., in lymphocytes, macrophages, mast cells, endothelial cells, and neurons (Mikkelsen et al. 2001; Yang et al. 2001, 2007; Pietzsch and Hoppmann 2009). Consequently, dysregulation in S100A12 secretion is found in many pathological situations like atherosclerosis, diabetes, chronic inflammatory disorders, and cancer (Kosaki et al. 2004; Salama et al. 2008; Mori et al. 2009; Pietzsch and Hoppmann 2009; Donato et al. 2013). The most extensively studied interaction partner of extracellular S100A12 is the proinflammatory receptor for advanced glycation endproducts (RAGE) (Hofmann et al. 1999; Leclerc et al. 2009; Hoppmann et al. 2010). In vitro findings in mast cells that did not show RAGE synthesis provided evidence on putative G-protein coupled S100 receptors (Yan et al. 2008). Moreover, in vitro experiments with endothelial cells and macrophages and in vivo experiments in rats using ^18^F-radiolabeled S100A12 provided first evidence that S100A12 also binds to scavenger receptors as demonstrated by us (Hoppmann et al. 2010). In this former study, scavenger receptor binding of S100A12 could be substantially blocked by maleylated bovine serum albumin (malBSA), a pan-scavenger receptor ligand, but not by polyinosinic acid and fucoidan, two class A scavenger receptor ligands (Acton et al. 1994; Platt et al. 1996; Fyrnys et al. 1997; Hsu et al. 2001; Thelen et al. 2010; Hoppmann et al. 2010; Yu et al. 2012). Of importance, malBSA binds to scavenger receptors of class A (SR-A1, SR-A2), class B (CD36), and the lectin-like oxidized low density lipoprotein (LDL) receptor-1 (LOX-1) (Sawamura et al. 2000; Mehta and Li 2002). This finding and further information from the literature predestined CD36 to be a strong candidate as S100A12-recognizing receptor of the scavenger receptor family. CD36 is a membrane receptor that is present on platelets, macrophages, monocytes, adipocytes, hepatocytes, myocytes, and some epithelial cells (Silverstein and Febbraio 2009). In monocytes and macrophages, CD36 acts as receptor and transporter for bioactive lipids leading to the activation of the peroxisome proliferator-activated receptor γ (PPARγ) signaling pathway, which subsequently regulates glucose metabolism and free fatty acid uptake (Tontonoz et al. 1998; Koonen et al. 2005; Graessler et al. 2007). It has to be considered that RAGE and certain scavenger receptors share a common ligand recognition principle based on electrostatic interactions between the positively charged receptor surface domains and negatively charged ligands (Adachi and Tsujimoto 2006; Jimenez-Dalmaroni et al. 2009; Gao et al. 2010; Fritz 2011). In this regard, there is an experimental evidence that well-characterized RAGE-ligands, such as advanced glycation endproducts, hypochlorite-modified proteins/apolipoproteins, and amyloid-β, also bind to CD36 (Ohgami et al. 2001; Kopprasch et al. 2004; Marsche et al. 2007; Jones et al. 2013). Another important property RAGE and CD36 have in common is the activation of signaling cascades (Goyette et al. 2009; Park 2014). This distinguishes CD36 from other scavenger receptors, e.g., those of class A. Additionally, S100A12 and CD36 are expressed in similar cell types, and more interestingly, the dysregulation of S100A12 and CD36 is related to similar pathological outcomes like atherosclerosis (Goyette et al. 2009; Park 2014).

To check the hypothesis that CD36 is a putative S100A12 receptor, this study aimed to investigate the possible interaction of S100A12 and CD36 on molecular and cellular level by the use of surface plasmon resonance (SPR), cell association and cell activation experiments.

## Methods

### Cell lines

Chinese hamster ovary (CHO)-K1 and CHO-CD36 cells were purchased from ATCC. CHO-RAGE cells were generated as described elsewhere (Hoppmann et al. 2010). Briefly, the vector pDNR-LIB-flRAGE was cloned into the eukaryotic expression vector pIRES-AcGFP1. For amplification, the vector was transfected into *E*. *coli* Top10 and positive clones were selected using 50 µg/mL kanamycin. Positive clones were checked for success of cloning and transfection using colony-PCR and sequencing analyses (Agowa GmbH). Transfection of plasmid-DNA into CHO-cells was performed using Lipofectamine™ 2000. Transfection was performed in serum free medium with 1 µg of plasmid-DNA and for 6–8 h at 37 °C under normal cell culture conditions (5 % CO_2_, v/v). Subsequently, cells were incubated in serum containing medium with selected antibiotics (300 µg/mL G418 and 250 µg/mL Zeocin). The success of transfection of CHO-RAGE cells was checked using fluorescence microscopy, Western blot analyses and flow cytometry.

### Immunocytochemical analysis

Synthesis of CD36 and RAGE was detected by immunocytochemical staining. Therefore, cells were fixed with 4 % (w/v) paraformaldehyde and 2.5 % (w/v) sucrose in phosphate buffered saline (PBS). After permeabilization with 0.3 % (v/v) Triton-X-100 in PBS, unspecific binding sites were blocked with a blocking solution containing 5 % (w/v) bovine serum albumin (BSA) and 0.5 % (v/v) Tween 20 in PBS. For antibody staining, the monoclonal anti-CD36 antibody [FA6-152] (abcam17044, 1:50) and the polyclonal anti-RAGE antibody (R&D systems, AF-1145 1:50) as well as secondary antibodies, anti-mouse AlexaFluor488^®^ (for anti-CD36 antibody) and anti-goat AlexaFluor594^®^ (for anti-RAGE antibody) were used. Counterstaining was conducted using the cell DNA marker Hoechst 33258 (5 µg/mL). Images were acquired using the confocal laser-scanning microscope IX83 (Olympus).

### Western blot analysis

Western blot analyses were performed as published elsewhere (Wolf et al. 2011). In short, sodium dodecyl sulfate–polyacrylamide electrophoresis of cell lysates with following semidry Western blotting was performed. Blots were blocked using blocking solution containing 5 % (w/v) dry milk powder, 2 % (w/v) BSA and 0.05 % (v/v) Tween 20 in Tris-buffered saline. For antibody staining, the monoclonal anti-CD36 antibody [FA6-152] (abcam17044, 1:500) and the polyclonal anti-RAGE antibody (R&D systems, AF-1145 1:500) as well as the peroxidase (POD) coupled secondary antibodies (anti-mouse IgG-POD for anti-CD36 antibody and anti-goat IgG-POD for anti-RAGE antibody) were used. Images were acquired using the Super Signal Dura and Pico kit (Thermo Scientific). Western blots were obtained from two experimental settings: (a) lysates were obtained from cells grown in cell culture medium supplemented with 10 % fetal calf serum, (b) for cell activation studies lysates were obtained from cells after incubation for 90 min with serum-free calcium binding buffer (20 mM 2-[4-(2-hydroxyethyl)piperazin-1-yl]ethanesulfonic acid (HEPES), 150 mM NaCl, 1.2 mM MgCl_2_, 1.3 mM CaCl_2_; pH 7.5).

### Recombinant S100A12 synthesis

Recombinant expression and purification of S100A12 (rS100A12) was performed as published elsewhere (Hoppmann et al. 2008). Briefly, pGEX-S100A12 transformed *E.coli* BL21 was cultivated in LB medium supplemented with 50 µg/mL ampicillin at 37 °C with shaking. When optical density (600 nm) of 1.0 was reached, protein expression was induced by adding 0.5 mmol/L isopropyl-β-d-1-thiogalactopyranoside (IPTG) for 4 h at 25 °C. The cell pellet was lysed using 20 % (v/v) Triton X-100, 4000 U/mL lysozyme, 25 U/mL benzonase and ultrasound. RS100A12 protein was purified using glutathione-sepharose. Glutathione *S*-transferase (GST) free proteins were eluted using PreScission™ protease (GE Healthcare). Further purification was performed using size exclusion chromatography. The purified protein was analyzed by mass spectrometry and Western blot (polyclonal goat anti-hEN-RAGE/S100A12, R&D Systems, AF1052). After snap freezing in liquid nitrogen, the purified protein was stored at -65 °C until usage for further experiments. Furthermore, commercially available native human S100A12 protein (nS100A12) was obtained from Life Technologies with the reference number 11143-HNAE-50. Compared to the purchased native nS100A12 the recombinant synthesized protein rS100A12 possesses five additional *N*-terminal amino acids (GPLGS) (Hoppmann et al. 2008).

### Fluorescence- and radiolabeling of recombinant rS100A12

Radiolabeling of rS100A12 with *N*-succinimidyl 4-[^18^F]fluorobenzoate ([^18^F]SFB) was performed as published by us earlier for the investigation of the interaction of S100A12 and RAGE (Hoppmann et al. 2008, 2010). Briefly, [^18^F]SFB diluted in acetonitrile was directly added to the rS100A12 solution in PBS and incubated for 30 min at 37 °C. The radiolabeled 4-[^18^F]fluorobenzoyl-([^18^F]FB)-rS100A12 was purified using a HiTrap desalting column (GE Healthcare) with the ÄKTAprime^®^ plus (GE Healthcare) chromatography system. The activity of the product was determined using the ISOMED 2000 calibrator (Nuklear-Medizintechnik Dresden GmbH).

To obtain a second, labeled S100A12 protein species, as independent marker for cell binding experiments and SPR studies (as described below), fluorescein-labeled rS100A12 was synthesized. Therefore, *N*-hydroxysuccinimide-fluorescein [(NHS)-fluorescein, Thermo Scientific] was used by following the manufacturer’s instruction. Briefly, 1 mg of rS100A12, (*M* = 10,974 g/mol, *c* = 1 mg/mL) in borate buffer (pH 8.0) was reacted with 15 times molar excess of NHS-fluorescein (*M* = 473.4 g/mol, *c* = 10 mg/mL) for 1 h at room temperature. Purification of the fluorescein-rS100A12 was performed in Slide-A-Lyzer™ dialysis cassettes (Thermo Fisher) with an exclusion size of *M* < 7000 g/mol. Depending on the following study PBS or calcium binding buffer (20 mM 2-[4-(2-hydroxyethyl)piperazin-1-yl]ethanesulfonic acid (HEPES), 150 mM NaCl, 1.2 mM MgCl_2_, 1.3 mM CaCl_2_; pH 7.5) were used.

### Cell binding experiments

To quantify the amount of [^18^F]FB-rS100A12 binding cells were incubated with [^18^F]FB-rS100A12 in PBS++ for 90 min at 37 °C. For blocking, a 15-fold excess of rS100A12 related to [^18^F]FB-rS100A12 or the monoclonal anti-CD36 antibody (FA6-152, abcam, *c* = 10 mg/mL) (Olivetta et al. 2014; Wang et al. 2014) was added to the cells 20 min at room temperature before starting the binding experiment. After incubation cells were lysed with NaOH (0.1 M) and sodium dodecyl sulfate (SDS) [1 % (w/v)]. Activity was quantified using the gamma counter COBRA™ II. Obtained activity values were put in relation to the starting activity (% ID) and the amount of protein in the cell lysate. Protein determination was performed using the Pierce^®^ bicinchoninic acid (BCA) Protein Assay (Thermo Scientific) following the manufacturer’s instruction. The amount of cellular fluorescein-rS100A12 binding was determined similar to the [^18^F]FB-rS100A12 experiments. Cells were grown in 24-well plates to 80 % confluence. Cells were incubated with 200 µL of incubation solution for 90 min at 37 °C. For antibody blocking experiments, the monoclonal anti-CD36 antibody (FA6-152, abcam) was added to the cells for 30 min at 37 °C in a concentration of 10 µg/mL before starting the cell binding experiment. Afterwards, cells were lysed with sodium hydroxide (NaOH, 0.1 M) and SDS [1 % (w/v)]. Fluorescence signal quantification was checked with assays using cell lysates using the Synergy™ 4 Multi-Mode Microplate Reader (BioTek) with *λ*_Ex_ = 485 nm, *λ*_Em_ = 525 nm as filter setting. Obtained relative fluorescence units were put in relation to the amount of protein in the cell lysate. Protein determination was performed using the Pierce^®^ BCA Protein Assay (Thermo Scientific) following the manufacturer’s instruction.

### Surface plasmon resonance

The SPR analyses were performed using a Biacore T100 (GE Healthcare). The ligand, CD36 (His-tagged recombinant human protein, Sino Biological, Life technologies, 70-80 kDa), was immobilized on a C1 sensor chip using the amine coupling kit (GE Healthcare) and PBS (GE Healthcare). The employed C1 amine coupling procedure comprises a surface cleaning step using a solution of 0.03 % (v/v) Triton T100 in 100 mM glycine (pH 12.3), the surface activation by 1-ethyl-3-(3-dimethylaminopropyl)carbodiimide (EDC)/NHS, the coupling step using a solution of CD36 at a concentration of 20 µg/mL in 10 mM acetate buffer (pH 4), and the blocking of the surface using ethanolamine. Afterwards 1000 RU of CD36 was immobilized on the sensor surface. A reference cell was prepared by blank immobilization. 350 µM CaCl_2_ in HEPES buffered saline (HBS)-P + buffer (GE Healthcare) was sterile filtered and used for further SPR experiments. Kinetic data were obtained by a single cycle kinetic using the respective S100A12 species rS100A12 and nS100A12 in concentrations of 0.62, 1.85, 5.56, 16.67, and 50.0 nM. The analyte was injected over the two flow cells at a flow rate of 30 µL/min and at a temperature of 25 °C. The association and dissociation time was 90 and 900 s, respectively. The surfaces were regenerated by a sequence of glycine (10 mM, pH 1.5, 5 s) and NaOH (0.05 mM, 5 s) followed by a stabilization period of 300 s. Starting from the respective solution of r/nS100A12, six 50 nM stock solutions were prepared which were further diluted in triplicate to yield the given final concentrations of the analyte. A buffer blank run was performed at the beginning and the end of each sequence comprising nine single cycle kinetics with r/nS100A12. Data were collected at a rate of 10 Hz. The data were fitted to a two-state reaction model including reference subtraction and blank buffer correction derived from both blank runs using the Biacore Evaluation software 2.0.4.

### Cell activation experiments

Cells were grown in 100 mm petri dishes or chamber slides to 80 % confluence. After short washing with PBS, cells were incubated with 2 % (w/v) BSA in calcium binding buffer for 1 h at 37 °C. Afterwards, incubation with 5 µM endotoxin free rS100A12 in calcium binding buffer or with calcium binding buffer alone, as negative control, was performed for 90 min at 37 °C. Cells were lysed for Western blot analysis or fixed using 4 % (w/v) paraformaldehyde for immunocytochemical staining. Western blot analysis of PPARγ synthesis was performed using a monoclonal antibody (abcam191407). Additionally, Western blot analyses of tyrosine kinases Fyn (#4023, cell signalling), pFyn (ab53690, abcam), Lyn (#2796, cell signalling), pLyn (ab33914, abcam) and mitogen-activated kinase p38 (ab4822, abcam) were performed.

### Statistical analysis

Statistical significance of all cell-binding experiments and SPR experiments was calculated using a one-way ANOVA followed by a Bonferroni post hoc test for the column analysis with Prism6 (GraphPad Software). Statistical significance was assumed for *p* < 0.05 and *p* < 0.01.

## Results

### CHO cell lines expressing CD36 and RAGE

**Fig. 1:**
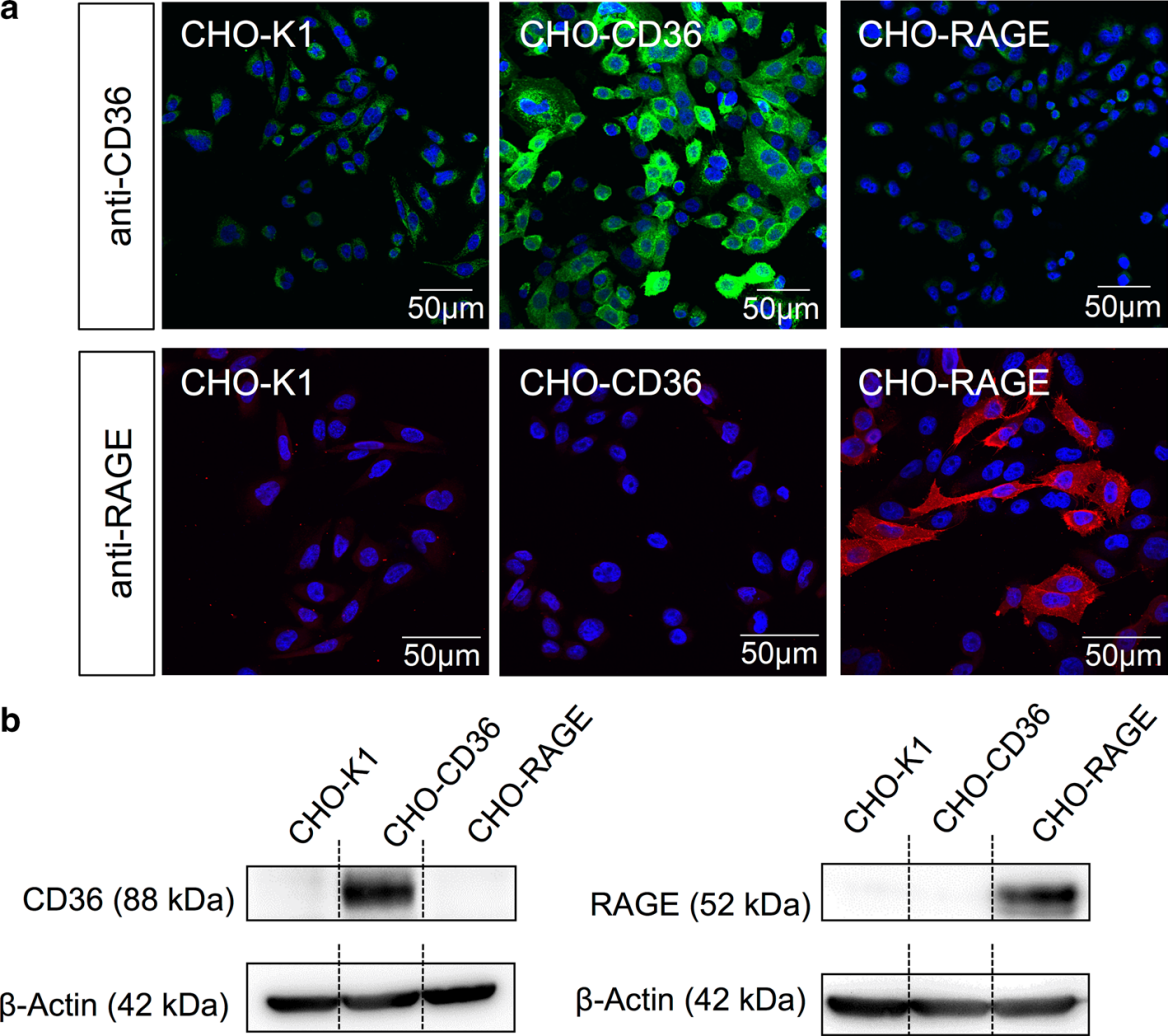
CD36 and RAGE expression. **a** Immunocytochemistry of CHO-K1, CHO-CD36 and CHO-RAGE cells using anti-CD36 (*green*) and anti-RAGE (*red*) specific antibodies. **b** Western blot analyses of CHO-K1, CHO-CD36 and CHO-RAGE cell lysates using anti-CD36 and anti-RAGE specific antibodies

Immunocytochemistry (Fig. 1a) and Western blot analyses (Figs. 1b, S1a) revealed the specific synthesis of the two receptors of interest by the different CHO cell lines. CHO-K1 showed no synthesis of RAGE in Western blot and immunocytochemistry. A very low synthesis of CD36 could be observed for the CHO-K1 cells. The CD36 synthesis could also be observed in an even smaller amount for the CHO-RAGE cells. On the other hand, the CHO-CD36 cell lines showed very high synthesis of CD36 in immunocytochemical images and with Western blot. Immunostaining for RAGE revealed that only the CHO-RAGE cell line showed synthesis of RAGE.

### Cell binding experiments

**Fig. 2:**
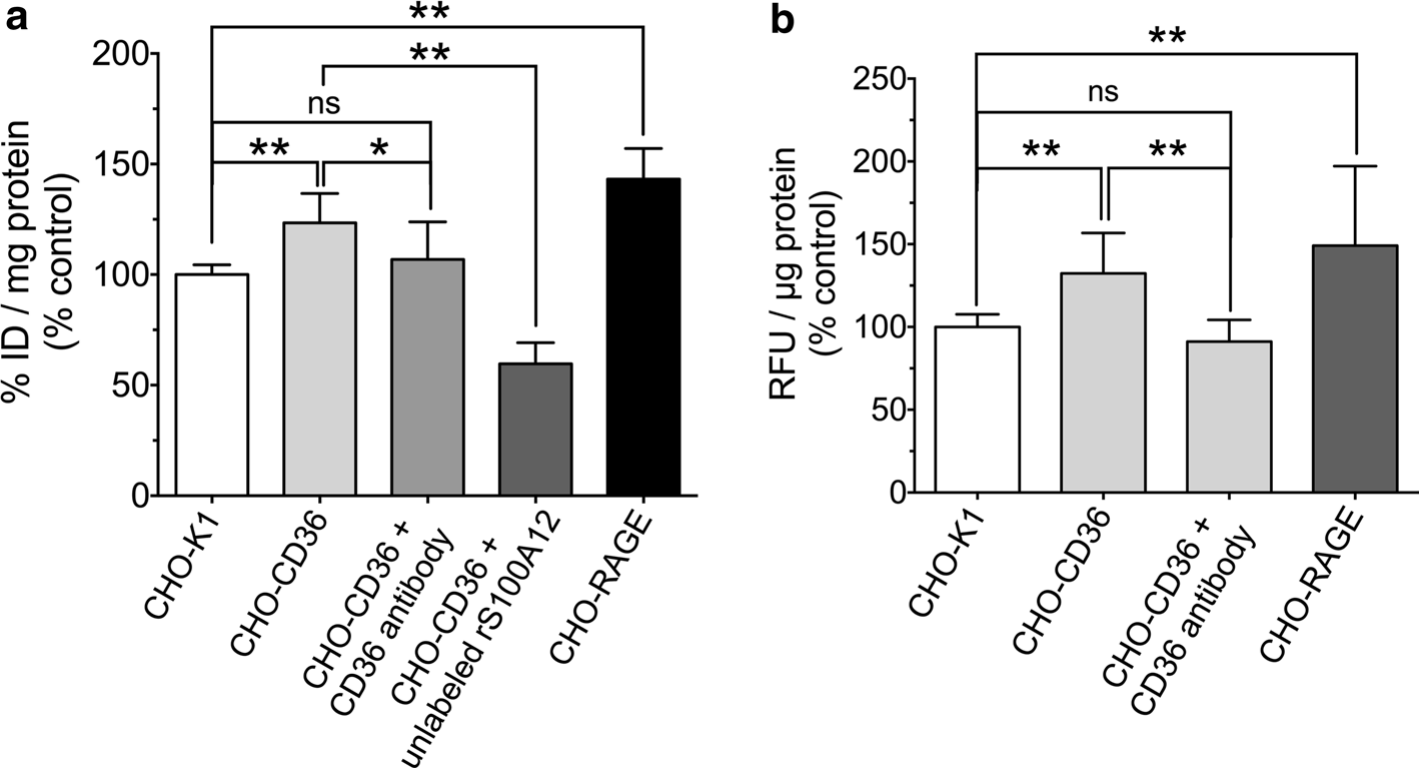
Cell binding of labeled rS100A12. Cell binding experiments with **a** [^18^F]FB-rS100A12 and **b** fluorescein-labeled rS100A12 were performed at 37 °C in CHO-K1 cells, CHO-CD36 cells, CHO-CD36 cells blocked with anti-CD36 antibody (and in case of **a** excess of unlabeled rS100A12), and CHO-RAGE cells. (*n* = 6–12, *<0.05, **p < 0.01)

In Fig. 2a the cell binding of [^18^F]FB-rS100A12 to the CHO cell lines at 37 °C is shown. Compared to CHO-K1 cells, cell association of [^18^F]FB-rS100A12 was significantly higher by 40 % in CHO-RAGE cells and by 20 % in CHO-CD36 cells. On the other hand, using the CD36-specific antibody (FA6-152) binding of [^18^F]FB-S100A12 to CHO-CD36 cells could be substantially blocked. Therefore, a specific binding of [^18^F]FB-S100A12 to CD36 could be confirmed. The fact that [^18^F]FB-rS100A12 was blocked by unlabeled rS100A12 indicates a similar binding behavior of both the unlabeled and the labeled species.

As independent marker for cell binding fluorescein labeled S100A12 was used. Therefore, experiments were performed with 10 µM of fluorescein-S100A12. Compared to CHO-K1 cells, binding was significantly higher by 50 % in CHO-RAGE and by 30 % in CHO-CD36 cells (Fig. 2b). Using a specific monoclonal blocking antibody (FA6-152) against CD36, binding to CHO-CD36 cells was reduced by 40 % compared to the non-blocked situation which indicates the specificity of the interaction.

### SPR analyses

**Fig. 3:**
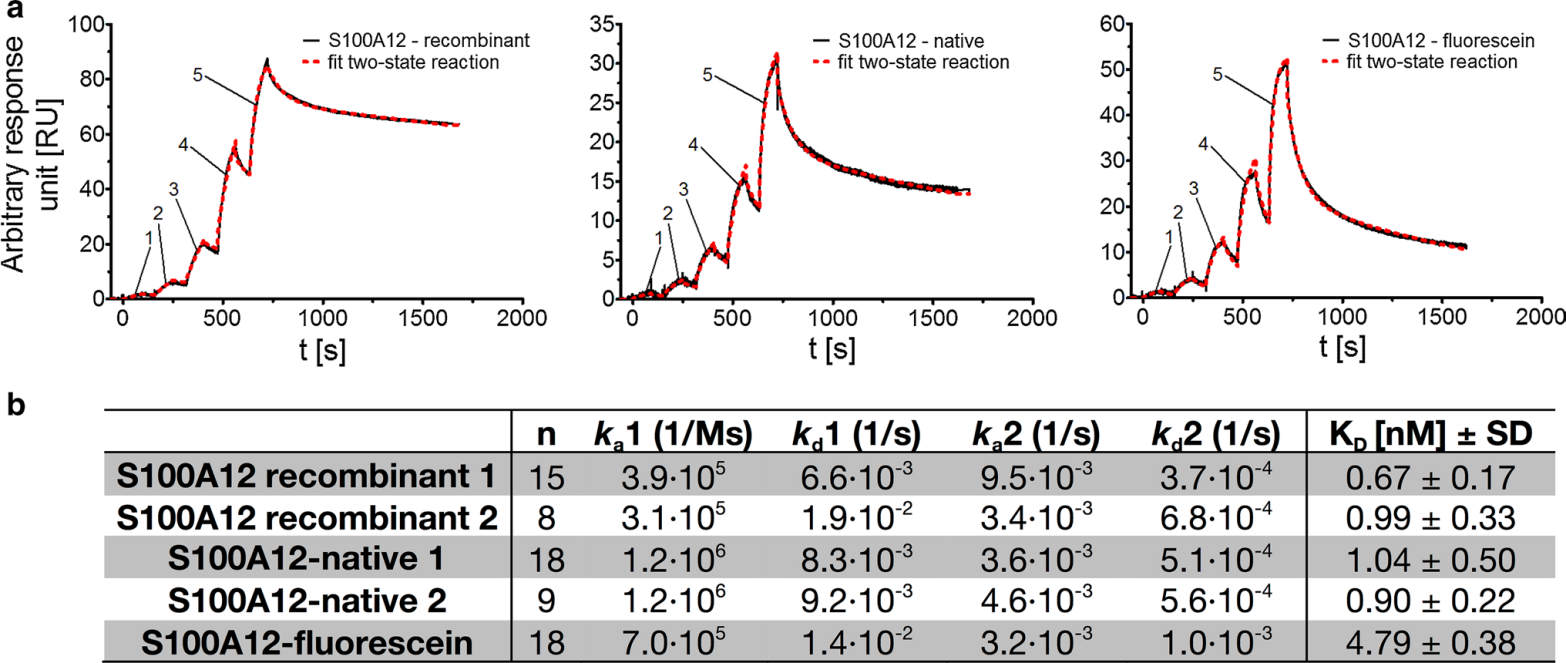
In vitro binding of S100A12 proteins to immobilized CD36. Surface plasmon resonance binding experiments of **a** rS100A12 (*left*), nS100A12 (*middle*), and fluorescein-labeled rS100A12 (*right*) to CD36 were performed. Results of representative experiments (subtracted sensorgrams) are shown. For evaluation of S100A12 binding kinetics, single-cycle kinetics was performed using concentrations ranging from 0.62 to 50.0 nM (1–0.62, 2–1.85, 3–5.56, 4–16.67, 5–50 nM). *Solid lines* represent observed data; *dotted lines* indicate computer-derived fits as calculated by a two-state reaction model. In **b** the calculated *k*_*a*_, *k*_*d*_, and, *K*_*D*_ values are shown. (*n* = 8–19)

SPR analyses were performed to characterize binding of S100A12 to CD36 at the protein–protein interaction level. Human recombinant CD36 (Life Technologies, reference number 10752-H08H-50, His Tag, active) was chosen as ligand and immobilized on the sensor surface by EDC/NHS amine coupling chemistry. A blank immobilized sensor surface served as reference cell to account for non-specific binding effects. Recombinant rS100A12, commercially available human, untagged recombinant nS100A12, and fluorescein-labeled rS100A12 were used as ligands. Experiments were performed as single cycle kinetics and analytes were used in a concentration range of 0.62–50.0 nM (Fig. 3a). *K*_D_ values were determined by fitting the observed sensorgrams using a two-state reaction model. The rS100A12, nS100A12, and fluorescein-labeled rS100A12 showed binding to CD36 (Fig. 3a, b). The affinities of rS100A12 (batch #1: *K*_D_ = 0.67 ± 0.17 nM; batch #2: *K*_D_ = 0.99 ± 0.33 nM) and commercially available nS100A12 (batch #1: *K*_D_ = 1.04 ± 0.50 nM; batch #2: *K*_D_ = 0.90 ± 0.22 nM) to CD36 did not differ significantly and were determined to be close to 1 nM. In comparison, fluorescein-labeled rS100A12 showed a weaker binding affinity (*K*_D_ = 4.79 ± 0.38 nM) to CD36.

### Cell activation experiments

**Fig. 4:**
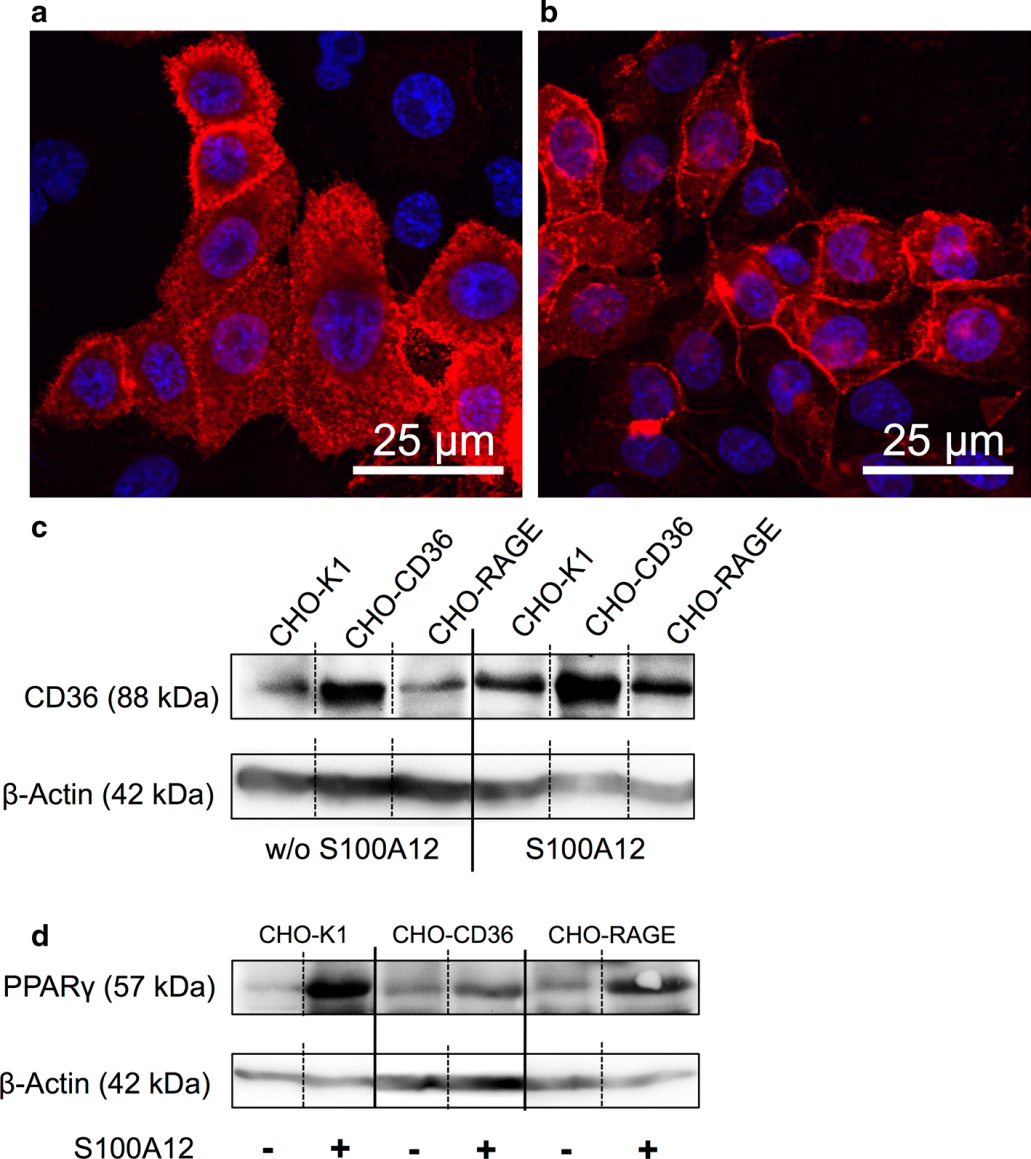
rS100A12 stimulated CD36 translocation, CD36 expression, and PPARγ expression. Immunohistochemical staining of CHO-CD36 cells for CD36 (*red*) **a** before and **b** after incubation with 5 µM of rS100A12, cell nuclei are stained with Hoechst 33258 (*blue*). Representative Western blot analysis of **c** CD36 synthesis (*n* = 5) and preliminary Western blot analysis of **d** PPARγ synthesis of CHO-K1, CHO-CD36, and CHO-RAGE cells without (−) and after activation (+) with 5 µM of rS100A12 in calcium binding buffer for 90 min

Although S100A12 showed high affinity to CD36, activation of proteins that interact directly with CD36 or are activated by the CD36 signaling cascade (Fyn, pFyn, Lyn, pLyn, p38) could not be observed in CHO-K1, CHO-CD36, and CHO-RAGE cells by immunoblotting (Fig. S2a–e). However, incubation of CD36-positive CHO cells with rS100A12 lead to the recruitment of CD36, which normally is diffusely distributed in vesicles, to the cell surface (Fig. 4a, b). On the other hand, the incubation of CHO-K1, CHO-CD36, and CHO-RAGE cells with rS100A12 lead to an increased synthesis of CD36 itself (Figs. 4c, S1b). This can be seen especially in CHO-K1 and CHO-RAGE cell lines that have low expression of CD36 under baseline conditions as already described above. Additionally, an up-regulation of the PPARγ synthesis of all cell lines could be observed in preliminary studies (Fig. 4d). Therefore, potential regulatory mechanisms based on binding of S100A12 to CD36, and subsequently, leading to both higher recruitment of CD36 to the cell membranes and an elevation of CD36 synthesis could be hypothesized.

## Discussion

For the investigation of potential S100A12–CD36 binding, CHO cell lines were used. Immunoblotting and immunocytochemical analyses revealed that the CHO-K1 cell line is a good negative control exhibiting no synthesis of RAGE and only low synthesis CD36. On the other hand, CHO-CD36 cells and CHO-RAGE cells show a high synthesis of CD36 and RAGE, respectively, without or with low synthesis of the respective other receptor. This study substantiates our former hypothesis on binding of S100A12 to members of the scavenger receptor family (Hoppmann et al. 2010) and first demonstrated class B scavenger receptor CD36 involved in high-affinity binding of S100A12. Cell binding experiments using both [^18^F] fluorobenzoylated and fluorescein-labeled rS100A12 showed that S100A12 specifically binds to cells expressing CD36. Similarly, although in a higher amount, [^18^F]FB-rS100A12 binds to cells expressing RAGE, a well-characterized receptor of S100A12. Blocking with a CD36-specific monoclonal antibody [FA6-152] that specifically blocks the collagen and thrombospondin binding sites of CD36 (Doebele et al. 2009), revealed that S100A12 most likely shares the same binding site as thrombospondin or collagen and that the binding is specific (Silverstein and Febbraio 2009). Importantly, it has been described that this binding site is not identical to the fatty acid binding site of CD36, which is important for its function as fatty acid transporter (Silverstein and Febbraio 2009). The in vitro cell binding experiments were confirmed by SPR analyses. The latter revealed subnanomolar to low nanomolar binding affinities for unlabeled r/nS100A12 and fluorescein-labeled rS100A12 to CD36.

Interestingly, the incubation of the different CHO cells with rS100A12 led to the recruitment of CD36 to the cell membranes. CD36 membrane recruitment is known to be regulated also by other stimuli like insulin (Samovski et al. 2012). Incubation of the CHO cells with S100A12 also leads to an increase of CD36 synthesis. This effect is even more striking looking at the less CD36 expressing cells like CHO-K1 and CHO-RAGE. Even though this effect could be due to the autoregulation of CD36 it could also be caused by the binding of S100A12 to RAGE (Xanthis et al. 2009). This would explain the high up-regulation of CD36 in CHO-RAGE cells but not for the RAGE-negative CHO-K1 and CHO-CD36 cells. This finding leads to the presumption that S100A12 could activate PPARγ through increased import of bioactive lipids (Ahmadian et al. 2013) through a not completely understood pathway (Silverstein 2009), which is consecutively increased by the membrane recruitment and the increase of CD36 synthesis (Tontonoz et al. 1998). The activation of PPARγ could also be seen in the higher protein synthesis of the protein itself.

Our results confirm that S100A12 shares the same binding site on CD36 as thrombospondin-1/-2 and collagen between amino acids 93 and 120 of CD36 (Doebele et al. 2009; Silverstein and Febbraio 2009). Oxidized LDL, on the other hand, has another binding site at the CD36 protein (Silverstein and Febbraio 2009). This is likely to support the hypothesis that the observed interaction of S100A12 and CD36, the subsequent recruitment of CD36 to the membrane, and the up-regulation in CD36 synthesis, could be involved in translocation of fatty acids and regulation of PPARγ (Kopprasch et al. 2004; Graessler et al. 2007; Silverstein and Febbraio 2009). But to prove this hypothesis, further investigations have to be carried out. Another hypothesis that could be deduced from our data will attribute participation of CD36 in forming clusters of various pattern recognition receptors, including RAGE and/or Toll-like receptors (Drage et al. 2009; Jimenez-Dalmaroni et al. 2009; Xanthis et al. 2009; Jones et al. 2013). Here, CD36 could contribute exclusively to high-affinity binding of S10012 without activation of CD36-specific downstream signaling, but activation of signaling pathways downstream the suspected individual co-receptors. A future challenge will be the experimental differentiation of the conceivable, often highly redundant pathways (Lin 2006).

## Concluding remarks

Taken together this study revealed S100A12 as potent binding partner of the class-B scavenger receptor/fatty acid translocase CD36. Binding could be observed by cell association experiments of labeled S100A12. SPR confirmed the binding revealing affinities in the low nanomolar range. A recruitment of CD36 to the surface of the CHO-CD36 cells could be observed, which suggest a regulatory function of S100A12 for the lipid transport by direct interaction with CD36.

## Electronic supplementary material

Below is the link to the electronic supplementary material.

Supplementary material 1 (DOCX 3581 kb)

## Abbreviations

[^18^F]FB: 4-[^18^F]fluorobenzoyl-
[^18^F]SFB: *N*-succinimidyl-4-[^18^F]fluorobenzoate
BCA: Bicinchoninic acid
BSA: Bovine serum albumin
CHO: Chinese hamster ovary
EDC: 1-Ethyl-3-(3-dimethylaminopropyl)carbodiimide
GST: Glutathione *S*-transferase
HEPES: 2-[4-(2-hydroxyethyl)piperazin-1-yl]ethanesulfonic acid
HBS: HEPES buffered saline
IPTG: Isopropyl-β-d-1-thiogalactopyranoside
LDL: Low density lipoprotein
LOX: Lectin-like oxidized low density lipoprotein (LDL) receptor
malBSA: Maleylated bovine serum albumin
NHS: *N*-hydroxysuccinimide
PBS: Phosphate buffered saline
POD: Peroxidase
PPAR: Peroxisome proliferator-activated receptor
RAGE: Receptor for advanced glycation endproducts
SDS: Sodium dodecyl sulfate
SPR: Surface plasmon resonance
