# Supplementary material for: Insights into binding of S100 proteins to scavenger receptors: class B scavenger receptor CD36 binds S100A12 with high affinity
Source: Amino Acids. 2016 Oct 12;49(1):183–91. doi: 10.1007/s00726-016-2349-2 (PMC5241339; doi:10.1007/s00726-016-2349-2)
Supplement: Supplementary file 1 — Supplementary material 1 (DOCX 3581 kb) [file 726_2016_2349_MOESM1_ESM.docx]

# *Supplementary material*

Insights into binding of S100 proteins to scavenger receptors: class B scavenger receptor CD36 binds S100A12 with high affinity

Christoph Tondera^1,2^, Markus Laube^1^, Jens Pietzsch^1,2,*^

^1^Helmholtz-Zentrum Dresden-Rossendorf, Institute of Radiopharmaceutical Cancer Research, Department of Radiopharmaceutical and Chemical Biology, Dresden, Germany

^2^Technische Universität Dresden, Department of Chemistry and Food Chemistry, Dresden, Germany

*Corresponding author: Tel.: +49 351 260 2622. j.pietzsch@hzdr.de (J. Pietzsch)


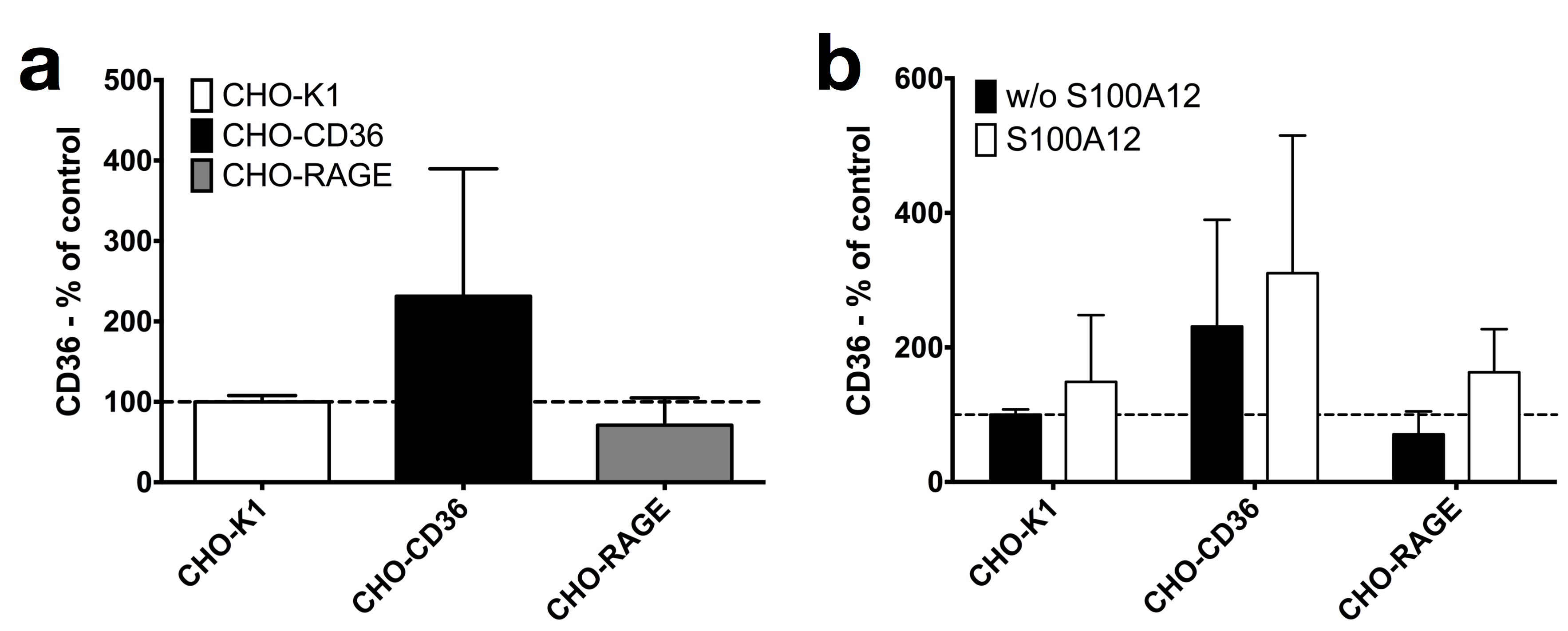


Figure S1: Western blot analyses of CD36 synthesis. Densitometric analyses of CD36 Western blots for (a) CHO-K1, CHO-CD36, CHO-RAGE without treatment (n=6) and (b) compared to S100A12 treatment (n=3), mean + S.D. After gel analysis was performed using background subtraction and automated band detection, values for the area under the curve were calculated for CD36 and actin (Total Lab software). Afterwards, synthesis of CD36 in relation to actin was calculated. Control cell line CHO-K1 was set to 100%. CD36 synthesis values are given as % of control cell line.


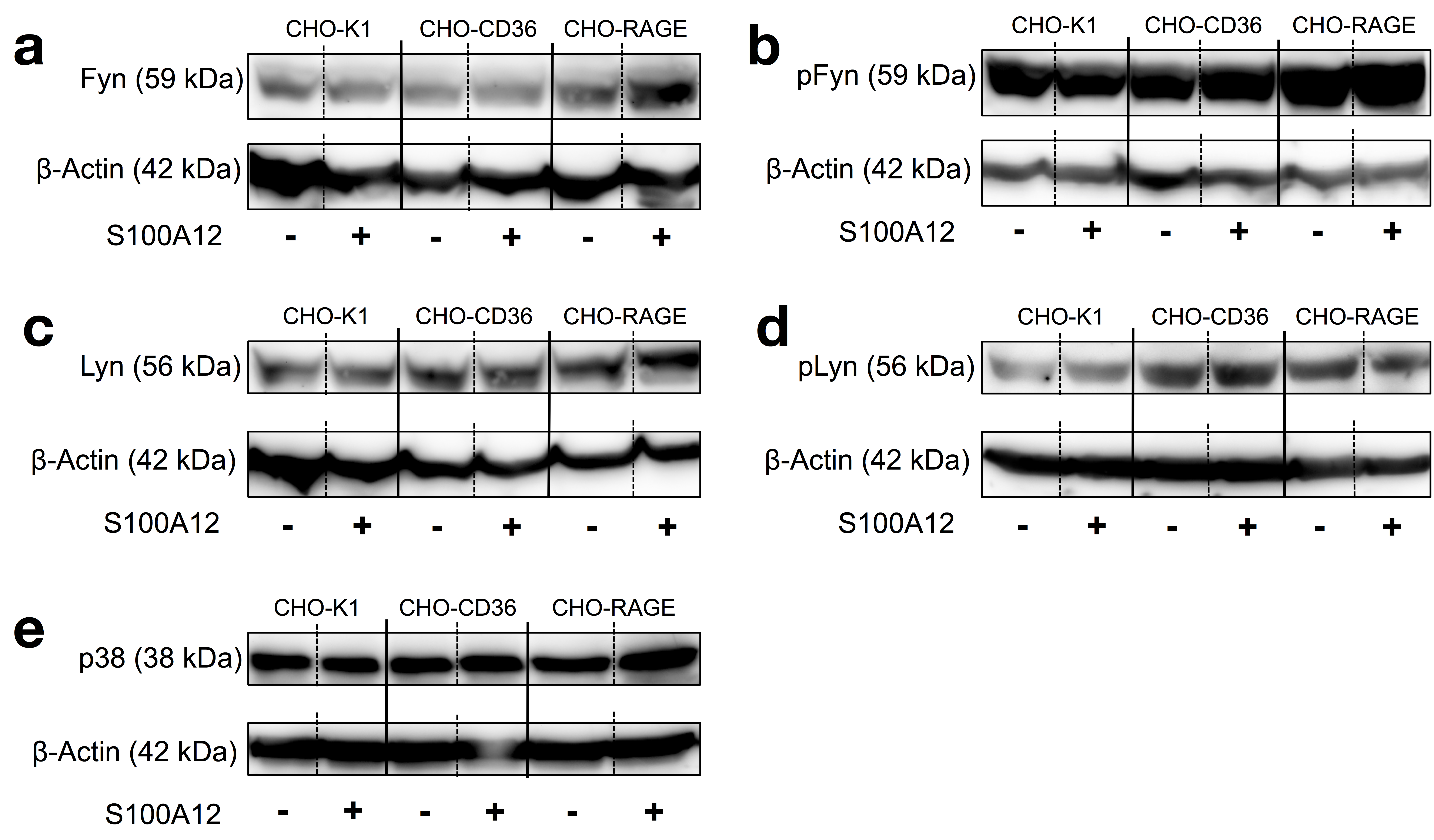


Figure S2: Cell activation experiments. Western blot analyses of tyrosine kinase (a) Fyn, (b) phosphorylated Fyn (pFyn), (c) Lyn, (d) phosphorylated Lyn (pLyn), and mitogen-activated kinase (e) p38 synthesis in CHO-K1, CHO-CD36, and CHO-RAGE cells is shown without (-) and after activation (+) with 5 µM of S100A12 in sodium binding buffer for 90 min. Cells without rS100A12 incubation were incubated in sodium binding buffer alone for 90 min.
